# IRES-like element-mediated translation of vsp1S4(-) suppresses BmCPV replication via RNAi antagonism

**DOI:** 10.1371/journal.ppat.1014402

**Published:** 2026-07-24

**Authors:** Xialing Chen, Qian Teng, Haoni Xue, Lixuan Li, Xiaoyan Du, Min Zhu, Xing Zhang, Chengliang Gong, Xiaolong Hu

**Affiliations:** 1 School of Life Sciences, Soochow University, Suzhou, China; 2 School of Chemistry and Life Sciences, Suzhou University of Science and Technology, Suzhou, China; State key laboratory of Plant Environmental Resilience, College of Biological Sciences, China Agricultural University, CHINA

## Abstract

Double-stranded RNA (dsRNA) viruses are thought to express proteins exclusively from their sense strand, while the antisense strand serves primarily as a replication template. Whether the antisense strand harbors hidden coding potential remains largely unexplored. Here, by integrating ribosome profiling and mass spectrometry, we identify a conserved 78-amino acid microprotein, vsp1S4(-), encoded by an antisense small open reading frame (sORFs) of the *Bombyx mori* cypovirus (BmCPV) genome. We demonstrate that vsp1S4(-) translation is driven by a previously unrecognized IRES-like element. Functional characterizations reveal that vsp1S4(-) localizes to the plasma membrane and acts as a negative regulator of viral replication. Mechanistically, vsp1S4(-) interacts directly with the viral RNAi suppressor NSP8, competitively disrupting the NSP8-AGO2 complex. This action restores the host’s antiviral RNAi response, thereby limiting viral proliferation. Our findings challenge the conventional view of dsRNA virus coding capacity, unveil a novel viral immune evasion and replication control mechanism, and highlight antisense-encoded microproteins as potential targets for antiviral therapy.

## Author summary

DsRNA viruses are traditionally believed to produce proteins only from their sense strand, leaving the antisense strand as a mere replication template. However, whether the antisense strand hides additional protein-coding potential has remained unknown. In this study, we discovered 78 amino acids peptide—called vsp1S4(-), which is encoded by the antisense strand of BmCPV, a dsRNA virus that infects silkworms. We found that this microprotein is produced by an unusual internal translation mechanism. Once there, it acts as a brake on viral replication by interfering with a viral NSP8, which normally blocks the host’s natural antiviral defense system (known as RNA interference). By binding to NSP8, vsp1S4(-) frees the host’s defense machinery to fight back, thereby limiting viral spread. Our results overturn the long-standing assumption that dsRNA virus antisense strands are non-coding, reveal a new layer of viral self-regulation, and suggest that such hidden viral proteins could be promising targets for developing antiviral strategies.

## Introduction

Viruses, which are constrained by compact genomes as noncellular entities, have evolved sophisticated strategies to expand their coding capacity, notably through the utilization of small open reading frames (sORFs) [1–6]. Recent studies have identified functional microproteins derived from sORFs within viral genomes [7,8]. For example, the Golgi-localized V3 microprotein encoded by geminiviruses suppresses host RNA silencing [2]. Similarly, a microprotein encoded by the antisense strand of turnip mosaic virus (TuMV, + ssRNA) localizes to perinuclear punctate granules and is essential for viral replication [9]. Furthermore, microproteins translated from upstream open reading frames (uORFs) in the Zika virus modulate neurotropism within human brain organoids [10,11]. Notably, microproteins encoded by overlapping ORFs (rORFs) on the SARS-CoV-2 antisense strand antagonize type I interferon signaling [9]. These viral microproteins modulate critical host cellular processes, regulate viral replication, and significantly contribute to pathogenesis.

*Bombyx mori* cypovirus (BmCPV) is a double-stranded RNA (dsRNA) virus that belongs to the genus *Cypovirus* within the family *Reoviridae*, specifically infecting silkworm midgut tissue [12]. Its genome comprises ten segments (S1-S10), encoding structural proteins (VP1, VP3, VP4, VP6, and VP7) and the RNA-dependent RNA polymerase VP2/RdRp, as well as nonstructural proteins (NSP5, NSP8, NSP9, and polyhedrin) [13–15]. These proteins orchestrate critical processes, including host cell invasion [16], virion assembly [17], and viral proliferation [18]. Despite their recognized multifunctionality, it remains uncertain whether these ten canonical proteins fully account for productive infection. Recent research highlights the crucial role of viral microproteins, often derived from non-canonical open reading frames (ORFs), as key mediators of pathogen-host interactions. Examples include microproteins translated from viral circular RNAs (circRNAs), such as vSP21, which modulates NF-κB signalling and autophagy [19]. And the S10 segment that produces a mitochondrial-targeting microprotein, VSP59, which exerts antiviral activity by activating prohibitin 2-mediated apoptotic pathways [20]. However, the functional potential of proteins encoded by the antisense (-RNA) strands of the BmCPV dsRNA genome remains largely unexplored.

In this study, to systematically address the knowledge gap, we employed an integrated multiomics approach combining ribosome profiling (Ribo-seq) and liquid chromatography-tandem mass spectrometry (LC-MS/MS) to decode the cryptic proteome originating from the BmCPV genome. This strategy led to the discovery of vsp1S4(-), a novel 78-amino acid microprotein encoded by an antisense ORF of segment 4. We show that vsp1S4(-) is translated via an internal ribosome entry site (IRES)-like element and localizes to the plasma membrane as a structural component of viral particles. Crucially, we demonstrated that vsp1S4(-) physically interacts with the viral RNA interference (RNAi) suppressor NSP8, thereby disrupting the NSP8-AGO2 complex, restoring host RNAi activity, and ultimately suppressing viral replication. Thus, vsp1S4(-) represents a viral self-limiting factor that counteracts the immunosuppressive function of NSP8. Collectively, our findings reveal the substantial, previously unrecognized coding capacity embedded within the antisense strands of a dsRNA viral genome and underscore the critical roles of BmCPV-derived microproteins in modulating antiviral innate immune responses.

## Results

### Unveiling a hidden repertoire of microproteins encoded by the BmCPV dsRNA genome

**Fig 1:**
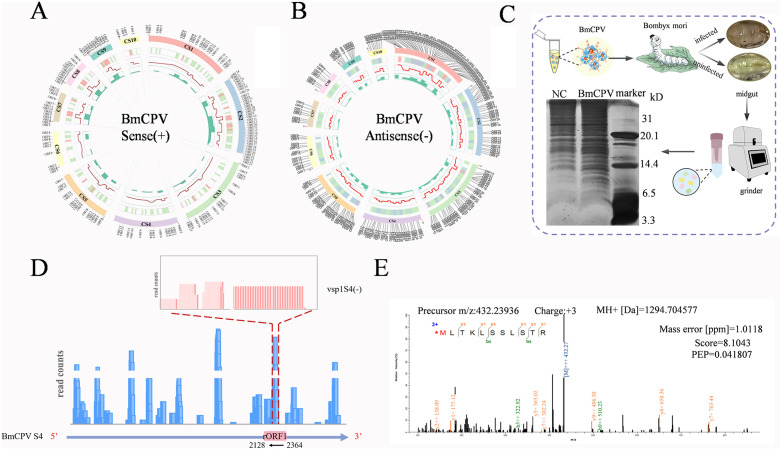
Identification and validation of BmCPV-encoded microproteins. **(A-B)** In silico prediction of sORFs in the BmCPV dsRNA genome. **(A)** Sense-strand (+RNA) sORF distribution (148 candidates) and (B) antisense-strand (-RNA) sORF distribution (237 candidates), which encode peptides of 20-100 amino acids, were identified via NCBI ORFfinder. Outermost ring: Segments 1-10 of BmCPV, color-coded; length represents segment size. Second ring: Heatmap. Third ring: Line plot. Fourth ring: Bar plot. **(C)** SDS-PAGE (silver-stained) results of ultrafiltration midgut proteins (<30 kDa). BmCPV particles (MOI = 2) infected the midgut tissue of 3-year-old silkworms. NC: uninfected midgut protein of silkworms; BmCPV: infected midgut protein of silkworms. **(D-E)** Translational evidence for vsp1S4(-). **(D)** Ribosome footprinting (Ribo-seq) of S4 (blue) and rORF1 (pink) read counts revealed their translational activity levels. **(E)** LC-MS/MS mapping of vsp1S4(-). MH+[Da]: Molecular weight of peptide segment; Charge: Electric charge; m/z: Mass-to-charge ratio of peptide.

Viral microproteins encoded by sORFs are increasingly recognized as critical regulators of viral replication, host-virus interactions, and pathogenesis [21–23]. To explore the microprotein-coding potential of the BmCPV dsRNA genome, we utilized the viral ORFfinder to analyze the BmCPV genome (Table 1). A candidate library was then generated for BLAST analysis (S1 Table). A total of 148 and 237 potential sORFs were identified within the sense (+RNA) and antisense (-RNA) regions of the BmCPV dsRNA genome, respectively, ranging in size from 20 to 100 amino acids (Fig 1A and 1B).

**Table 1:** The microprotein-coding potential of the BmCPV dsRNA genome.

Segment	Genbank	length (nt)	amino acids (aa)	Molecular weight (kD)	name	location	Non- coding region (5’/3’)
1	GU323605	4189	1333	148	VP1(V1)/CSP	Capsid protein	39/148
		186 (77-262)	62	7.6	sORF	unknown	76/3937
2	GQ924586	3854	1225	139	VP2	RNA polymerase	77/99
3	GQ924587	3846	1239	140	VP3/Spike protein	Spike protein	40/86
4	GU323606	3262	1058	120	VP4 (V3)/TP	Tarpin	13/62
5	GQ294468	2852	881	101	NSP5 (NS1)	Non-structural protein	70/136
					[NSP5a (NS2), NSP5b (NS6)]		
6	GQ294469	1796	561	64	VP6 (V4)	Capsid protein	43/67
7	GQ150538	1501	448	50	VP7 (V5)\LPP	Protrusion protein	24/130
8	GQ150539	1328	390	44	NSP8 (p44)	Non-structural protein	37/118
9	GQ924588	1186	320	36	NSP9 (NS5)	Non-structural protein	74/149
10	GQ924589	944	248	28	Polyhedrin	Non-structural protein	41/156

To experimentally evaluate translational activity, we conducted ribosome profiling (Ribo-seq) on BmCPV-infected and uninfected BmN cells. Analysis of the predicted sORFs revealed 567 ORFs supported by ribosome-protected fragments. Genomic mapping revealed that 89% of these translated ORFs overlapped with annotated coding sequences (CDSs). In comparison, 3% corresponded to downstream overlapping (doORF), downstream (dORF), and upstream overlapping (uoORF) ORFs, and 1% mapped to annotated ORFs or upstream ORFs (uORFs) (S1A Fig).

We also performed targeted proteomic validation using midgut tissue from BmCPV-infected and mock-infected silkworm larvae. Proteins <30 kDa were enriched via ultrafiltration, separated via SDS-PAGE, and visualized via silver staining (Fig 1C). Mass spectrometry (LC-MS/MS) analysis of gel bands below 14.4 kDa identified 17 unique microprotein-derived peptides (S2 Table). Crucially, five peptides originated from BmCPV antisense (-RNA) strands, with their spectra matching entries in our integrated Ribo-seq/LC-MS/MS database (S2A, S2B Fig). These findings provide direct experimental evidence for the expression of antisense-encoded microproteins during viral infection.

Bioinformatic analysis of the predicted microproteins included predictions of transmembrane domains, internal ribosome entry site (IRES) potential, and N6-methyladenosine (m⁶A) sites (S3A Fig). Among these, a 78-amino acid microprotein, vsp1S4(-) (rORF1, nt 899–1135 of the S4 antisense strand (S4(-))) was predicted to contain a TM domain, an IRES-like element, and an m^6^A site. Ribosome footprinting and MS analysis confirmed active translation across the rORF1 locus (Fig 1D, 1E).

Collectively, these findings reveal a previously unrecognized repertoire of microproteins encoded by the BmCPV dsRNA genome, highlighting the underappreciated coding capacity of antisense strands in dsRNA viruses.

### Validation of vsp1S4(-) expression and localization

**Fig 2:**
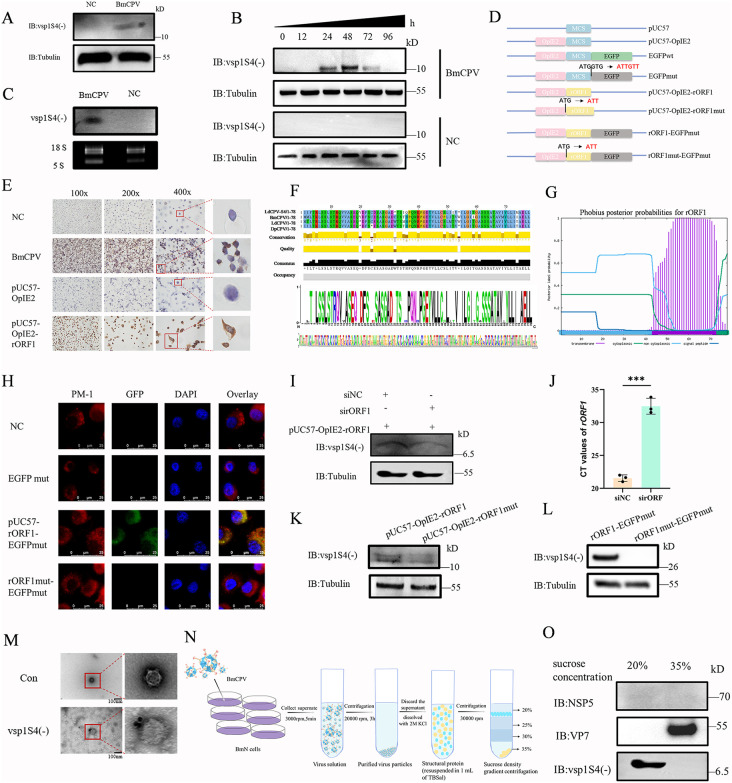
Expression, localization, and structural role of vsp1S4(-). **(A-F)** Validation of vsp1S4(-). (A) vsp1S4(-) was detected in infected midgut tissues. NC: uninfected; BmCPV: infected. **(B)** Western blotting analysis of vsp1S4(-) levels in BmN cells at indicated time points post-infection (0, 12, 24, 48, 72, 96 **h)**. Tubulin serves as loading control. **(C)** Northern blot analysis of total RNA (20 μg per lane) from BmN cells harvested at 48 hours post-infection. Ethidium bromide-stained 18S and 5S rRNAs serve as loading controls. **(D)** Plasmid construction diagram. **(E)** Immunohistochemistry (ICC) shows that vsp1S4(-) is present in infected BmN cells. **(F)** Multiple sequence alignment of vsp1S4(-) in different cypoviruses. ClustalW, Jalview, and Weblog were used to perform multiple sequence alignments. **(G-H)** Subcellular localization. **(G)** TM domain (51 to 73 aa) in vsp1S4(-) predicted by the TMHMM website. **(H)** Colocalization of vsp1S4(-) with the plasmalemma marker (PM-1) (red), antibodies against the identified GFP (green), and nuclei were stained with DAPI (blue). Scale bar: 25 μm. **(I-L)** Knockdown validation. **(I)** BmN cells transfected with siNC or sirORF1 were subjected to IB analysis. siNC: non‑targeting negative control siRNA; sirORF1: vsp1S4(-)-specific siRNA. **(J)** Quantitative analysis of rORF1. Data are presented as mean ± SD (***P < 0.01, n = 3). **(K and L)** Effects of mutation of rORF1 on the expression of vsp1S4(-). **(M-O)** Structural association with virions. **(M)** Immunogold detection of vsp1S4(-). TEM images showing immunogold labeling in control (upper) and experimental (lower) samples. Scale bar: 100 nm. **(N)** Diagram illustrating the separation procedure for vsp1S4(-). **(O)** Western blotting was used to detect the viral components at each concentration after sucrose density gradient centrifugation.

To validate vsp1S4(-) expression, we raised a polyclonal antibody against a synthetic peptide (residues 2–12) (S3B, S3C Fig). In infected silkworm midgut, this antibody recognized a specific band of ~8.4 kDa, which was absent in uninfected controls (Fig 2A). Endogenous expression showed that vsp1S4(-) expression was detectable as early as 24 hours post-infection (hpi) and gradually increased to highest at 48 hpi (Fig 2B). Ectopic expression of vsp1S4(-) using pUC57-OpIE2-rORF1 resulted in a dose-dependent increase in protein levels (S4A Fig). To directly confirm the presence of the antisense transcript encoding vsp1S4(-), we performed Northern blot analysis using a DIG-labeled probe targeting the vsp1S4(-) coding region (237 bp). A specific band was detected in BmCPV-infected cells at approximately the expected size, whereas no signal was observed in mock-infected controls, confirming the transcription of the S4 antisense RNA (Fig 2C). Immunohistochemistry of infected BmN cells or cells transfected with the rORF1 constructs (Fig 2D) showed strong brown-yellow staining, whereas controls showed no signal. It further confirmed the presence of vsp1S4(-) in BmCPV-infected BmN cells (Fig 2E). Multiple sequence alignment revealed that vsp1S4(-) is conserved across BmCPV isolates, indicating evolutionary selection pressure (Fig 2F). Bioinformatic analysis using TMHMM predicted a transmembrane domain spanning amino acids 51–73 (Fig 2G), and Softberry analysis predicted plasma membrane localization (S3D Fig). To experimentally validate this, we performed immunofluorescence co-localization assays using EGFP-tagged vsp1S4(-) and the plasmalemma marker PM-1. The results showed that vsp1S4(-)-EGFP co-localized with PM-1 at the cell periphery, whereas EGFP alone exhibited a diffuse cytosolic distribution (Fig 2H), confirming its plasma membrane localization. To assess the functional requirement of vsp1S4(-) during infection, we performed siRNA-mediated knockdown. Transfection of BmN cells with vsp1S4(-)-specific siRNA (sirORF1) significantly reduced endogenous vsp1S4(-) expression compared to the non-targeting control siRNA (siNC) (Fig 2I-2J). Furthermore, mutation of the rORF1 start codon (ATG → ATT) abolished vsp1S4(-) expression, confirming the specificity of the antibody and the requirement of the intact ORF for protein production (Fig 2K-2L).

Immunogold labeling coupled with transmission electron microscopy revealed specific gold particle enrichment on the surface of purified BmCPV particles, whereas no labeling was observed in control samples, indicating that vsp1S4(-) is exposed on the virion surface (Fig 2M). To further characterize the association of vsp1S4(-) with BmCPV virions, purified virus particles were first treated with 2 M KCl, and the resulting precipitate was collected for sucrose density gradient centrifugation (20‑35%). Western blotting analysis of the gradient fractions revealed that vsp1S4(-) predominantly sedimented in the 20% sucrose layer, whereas the major capsid protein VP7 was mainly detected in the 35% sucrose layer (Fig 2N-2O). This distribution pattern is consistent with vsp1S4(-) being incorporated into mature virus particles.

To distinguish structural proteins from nonstructural proteins, purified virions were sequentially extracted with low‑salt buffer (50 mM NaCl) and high‑salt buffer (1 M NaCl), followed by centrifugation (S4B Fig). Western blotting analysis demonstrated that the nonstructural protein NSP8 was efficiently extracted by low‑salt buffer and did not co‑sediment with the virion pellet. In contrast, both vsp1S4(-) and the capsid protein VP7 remained in the high‑salt extract and the virion pellet fractions (S4C‑S4D Fig). Collectively, these results demonstrate that vsp1S4(-) is a structural component of the BmCPV virion, tightly associated with the capsid fraction, and can be distinguished from nonstructural proteins by differential salt solubility.

### vsp1S4(-) translation might be derived from a viral IRES-like element

**Fig 3:**
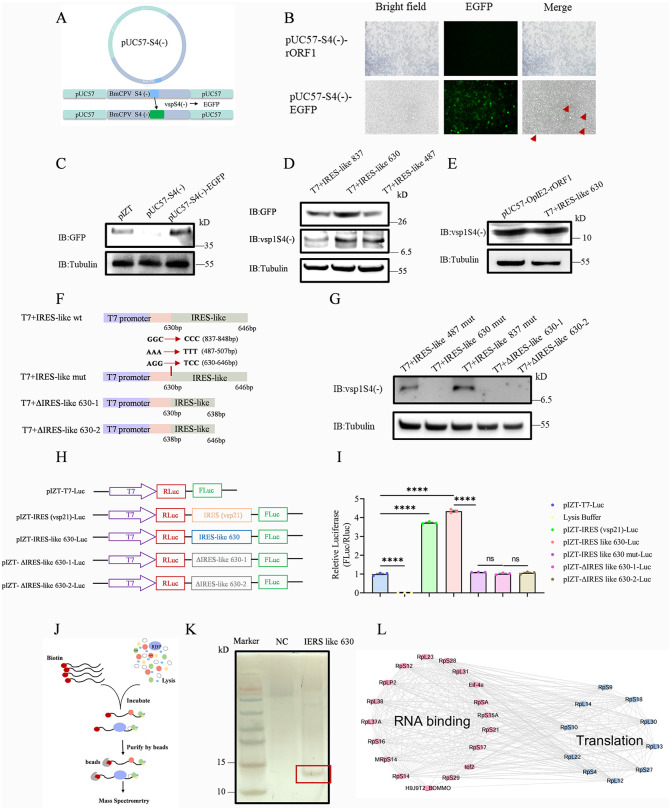
IRES-like element-mediated translation of vsp1S4(-). **(A-C)** IRES-like element activity assay. **(A)** Schematic of pUC57-S4(-)-EGFP. The rORF1 was placed by EGFP. **(B-C)** EGFP fluorescence and protein expression in transfected BmN cells. **(D-G)** IRES-like elements initiation translation of vsp1S4(-). **(D)** In vitro-transcribed IRES-like elements (487-507, 630-646, and 837-848 nt) were separately transfected into BmN cells for Western blotting. **(E)** Western blotting showing ectopic expression of vsp1S4(-) in BmN cells transfected with in vitro-transcribed RNA containing the T7 promoter and the IRES-like 630 element. **(F)** Diagram of *in vitro* transcription. **(G)** *In vitro* transcription assay: Truncated/mutated IRES-like drives vsp1S4(-) noncap-dependent translation. **(H)** Schematic representation of bicistronic reporter constructs. **(I)** BmN cells were co-transfected a series of pIZT-T7-IRES-Luc plasmids along with pRL-TK (Renilla luciferase) for normalization. Experimental: pIZT-IRES like 630-Luc, pIZT-IRES like 630 mut-Luc, pIZT-ΔIRES like 630 mut-1-Luc, pIZT-ΔIRES like 630 mut-2-Luc; pIZT-T7-Luc (control), pIZT-IRES (vsp21)-Luc (positive control), Lysis Buffer (negative control). Firefly luciferase (FLuc) activity was normalized to Renilla luciferase (RLuc) activity (mean ± SD, n = 3). ****P < 0.0001. **(J-L)** IRES-like 630 interactome. **(J)** Schematic diagram of RNA pull-down. **(K)** IRES-like 630 interactome. Probe of NC: ACCGCGACCGCGACCGCG; Probe of IRES-like 630: AGGGACAACAGATATA. **(L)** STRING analysis of 630 IRES-like trans-acting factors. RNA-binding (pink); translation (blue).

To investigate the mechanism by which the antisense-encoded vsp1S4(-) is translated, we first examined whether the 5′ untranslated region of the S4 antisense transcript contains cis-acting elements capable of initiating cap-independent translation. We constructed a reporter plasmid, pUC57-S4(-)-EGFP, in which the vsp1S4(-) open reading frame (rORF1) was replaced by *EGFP* (Fig 3A). Transfection of BmN cells with this construct resulted in detectable EGFP fluorescence and protein expression, suggesting that the S4 antisense transcript harbors internal translational control elements (Fig 3B-3C).

Bioinformatic analysis via the IRESite algorithm on the 500 bp upstream of the vsp1S4(-) start codon identified three high-confidence IRES-like sequences at positions 487–507 nt, 630–646 nt, and 837–848 nt (IRES-like 487, IRES-like 630, and IRES-like 837) (S5A Fig). To explore their translational activity, we performed in vitro transcription using primers containing a T7 promoter (Table 2) to generate RNA transcripts encompassing each IRES-like sequence (S5A Fig). We then transfected these RNA transcripts into BmN cells as EGFP fusion constructs. Western blotting analysis revealed robust EGFP expression better in cells transfected with the transcript containing IRES-like 630. Ectopic expression from a transfected T7-driven transcript containing IRES-like 630 vsp1S4(-) was confirmed by Western blotting, resulting in a specific band consistent with antisense strand translation (Fig 3D-3E).

**Table 2:** The primers used are listed in this study.

Primer name	Sequence (5’-3’)
T7 + IRES-like 487	TAATACGACTCACTATAGAAATAAGTATATAGAATTATT
T7 + IRES-like 837	TAATACGACTCACTATAGAGGCACTCGTAT
T7 + IERS-like 630	TAATACGACTCACTATAGAGGGACAACAG
T7 + IRES-like 630mut	TAATACGACTCACTATAG TCCCACAACAGATATA
T7 + ΔIRES-like 630–1	TAATACGACTCACTATAGAGGGACAA
T7 + ΔIRES-like 630–2	TAATACGACTCACTATAGCAGATATA
T7 + IRES-like 487mut	TAATACGACTCACTATAG TTTAAAGTATATAGAATTATT
T7 + IRES-like 837mut	TAATACGACTCACTATAG CCGACTCGTAT
R-EGFP	TTACTTGTACAGCTCGTCCATGC
R-rORF1	CAGTAATTCCGCAATGATTAATAGATACAT

To validate the specificity and requirement of IRES-like 630, we generated truncated and mutated versions of this element (Fig 3F). *In vitro*-transcribed RNAs carrying these mutations or deletions failed to drive vsp1S4(-) expression, demonstrating that the integrity of the 630–646 nucleotide sequence is essential for its IRES activity (Fig 3G). We next employed a bicistronic reporter system to quantitatively assess IRES activity. BmN cells were co-transfected with a series of pIZT-T7-IRES-Luc constructs together with pRL-TK (Renilla luciferase) for normalization. The experimental constructs included pIZT-IRES-like 630-Luc, pIZT-IRES-like 630 mut-Luc, pIZT-ΔIRES-like 630 mut-1-Luc, and pIZT-ΔIRES-like 630 mut-2-Luc, with pIZT-T7-Luc as a negative control and pIZT-IRES (vsp21) -Luc as a positive control (Fig 3H). Firefly luciferase activity was normalized to Renilla luciferase activity. The results showed that IRES-like 630 significantly enhanced luciferase expression compared to the control, whereas mutations or deletions of this element severely impaired its activity (Fig 3I). These data quantitatively confirm that the 630–646 region functions as a bona fide IRES-like element.To identify trans-acting factors governing cap-independent translation of vsp1S4(-), we employed the IRES-like 630 element as bait for affinity purification of cognate RNA-binding proteins (Fig 3J). Biotinylated IRES-like 630 RNA probes were used to capture RNA-protein complexes from BmN cell lysates via streptavidin bead enrichment. Liquid chromatography-tandem mass spectrometry (LC-MS/MS) analysis identified 40 high-confidence candidate proteins (Fig 3K, Table 3), whose interaction network was constructed using the STRING database (Fig 3L). Network clustering revealed two major functional modules: (1) ribosomal protein complexes, represented by members of the ribosomal protein L family (RpL) and S family (RpS), which are core components of the ribosomal large and small subunits, respectively; and (2) translation regulatory factors, including the translation initiation factor eIF4A and additional ribosomal proteins involved in translational machinery assembly. These findings suggest that the IRES-like 630 element orchestrates non-canonical translation by engaging a multiprotein complex comprising ribosomal subunits and translation factors, potentially bridging RNA processing and translational initiation machinery to facilitate cap-independent translation.

**Table 3:** Liquid chromatography-tandem mass spectrometry (LC-MS/MS) analysis identified high-confidence candidate proteins.

Sequence	Protein ID	Sequence	Protein ID
1	A0A8R2M424	21	Q5UAL6
2	A0A8R1WFG0	22	Q5UAM9
3	Q5UAN5	23	Q5UAN1
4	A0A8R1WH48	24	Q5UAM5
5	Q2F5L5	25	Q5UAN4
6	Q5UAS7	26	Q5UAM6
7	Q6TAC3	27	Q5UAP4
8	Q1HPK6	28	Q5UAM7
9	A0A8R2ARM1	29	Q5UAP7
10	Q1HPW2	30	Q5UAP8
11	A0A8R2M533	31	Q5UAQ7
12	A0A8R2R8E3	32	Q5UAP0
13	A0A8R2ASY4	33	Q5UAR6
14	A0A8R2ASW7	34	Q5UAR7
15	Q1HPY6	35	Q5UAS4
16	H9U396	36	Q5UAS6
17	Q5UAL3	37	Q5UAP9
18	Q1HPR1	38	Q6PS50
19	Q1EPM0	39	Q5UAQ8
20	Q5UAL8	40	Q6PUF8

### vsp1S4(-) inhibits virus replication

**Fig 4:**
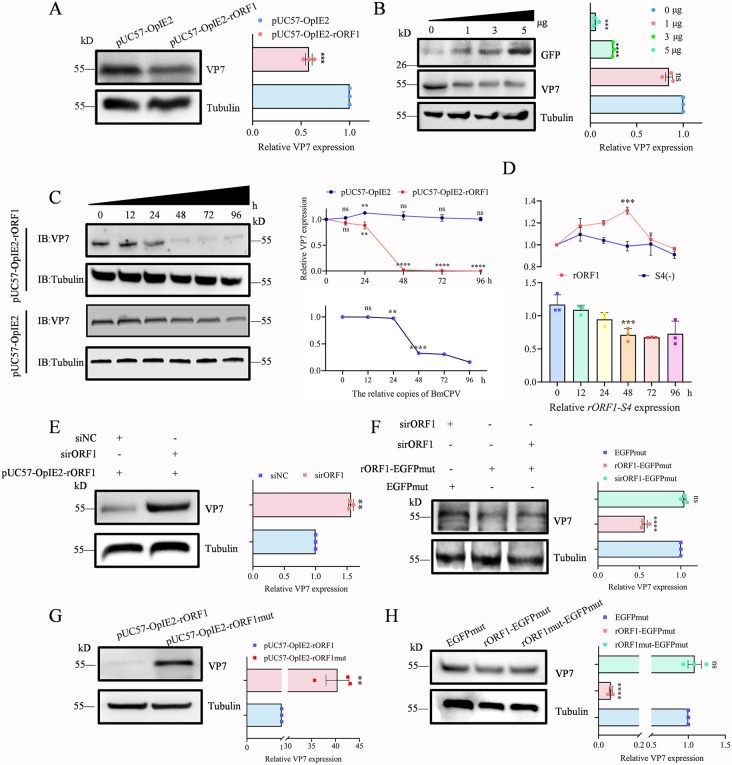
vsp1S4(-) inhibits BmCPV replication. (A-C) Dose- and time-dependent suppression. (A) vsp1S4(-) inhibited VP7 expression in pUC57-OpIE2-rORF1-transfected BmN cells (2 × 10⁶) compared with pUC57-OpIE2. (B) Dose-dependent vsp1S4(-)-mediated VP7 inhibition in rORF1-EGFPmut-transfected BmN cells (1/3/5 μg). (C) Time course of VP7 inhibition. Viral protein kinetics post vsp1S4(-) overexpression in BmN cells (0, 12, 24, 48, 72, and 96 h) vs. pUC57-OpIE2 (the left). The upper right panel shows the relative VP7 expression, while the lower right panel shows the relative BmCPV copy number. Data are mean ± SD (n = 3), ***P < 0.001. (D) BmN cells were transfected with pUC57-OpIE2-rORF1 (2 µg) or empty vector. Total RNA was extracted at 0, 12, 24, 48, 72, and 96 h post-transfection. real-time PCR was performed to measure S4(-) RNA levels, normalized to α-tubulin. Data are mean ± SD (n = 3), ***P < 0.001. (E) BmN cells were transfected with sirORF1 or siNC (20 nM). At 48 h post-transfection, cells were infected with BmCPV (MOI = 2). Western blotting analysis of VP7 protein level is shown. α-tubulin served as a loading control. (F) siRNA-mediated knockdown of vsp1S4(-) enhances viral protein VP7 expression. BmN cells were transfected with 20 nM of either a non-targeting control siRNA (siNC) or a vsp1S4(-)-targeting siRNA (sirORF1). At 24 hours post-transfection, cells were infected with BmCPV at an MOI of 2. Whole-cell lysates were collected 48 hours post-infection and analyzed by Western blotting using antibodies against VP7 and α-tubulin (loading control). The bar graph (right panel) shows the quantitative densitometric analysis of VP7 protein levels normalized to α-tubulin from three independent biological replicates (mean ± SD; **P < 0.01, ****P < 0.001, one-way ANOVA with Tukey’s post-hoc test). (G-H) Viral protein suppression requires an intact vsp1S4(-) open reading frame (rORF1). (G) BmN cells were transfected with wild-type rORF1 (WT) or start codon mutant (rORF1mut) plasmids, then infected with BmCPV (MOI = 2). VP7 and α-tubulin were detected by Western blotting. (H) BmN cells were transfected with constructs of EGFPmut, rORF1-EGFPmut, or rORF1mut-EGFPmut, then infected as above. EGFPmut alone serves as expression control. Bar graphs show relative VP7 levels normalized to α-tubulin from three independent replicates (mean ± SD). One-way ANOVA with Tukey’s post-hoc test. **P < 0.01; ***P < 0.001; ns, not significant.

Microproteins have emerged as essential regulators of viral replication, often exerting multiple effects on host-virus interactions (2, 9, 24). To define the function of the microprotein vsp1S4(-) in BmCPV infection, we investigated its effect on viral protein production. Compared with the control vector pUC57-OpIE2, the transfection of BmN cells with pUC57-OpIE2-rORF1 resulted in a dose-dependent reduction in the level of the major capsid protein VP7 (Fig 4A and 4B). Grayscale scanning confirmed decreased VP7 expression at higher vsp1S4(-) doses, demonstrating the negative regulation of viral structural protein synthesis by this dose.

This inhibitory effect was most pronounced within the first 48 hours post-transfection, coinciding with the early phase of viral protein production (Fig 4C). Consistent with the decrease in VP7, absolute quantification revealed a marked decrease in BmCPV genomic RNA copy number at 48 hours in vsp1S4(-)-expressing cells (Fig 4C). The expression of the parental gene was unaffected (Fig 4D). Furthermore, assessment of various IRES-like sequences revealed that IRES-like 630 has the most pronounced inhibitory effect on the expression of the viral protein VP7 (S5A, S5B Fig). This finding further indicates that IRES-like 630 acts as a key cis-acting element regulating vsp1S4(-).

The specificity of vsp1S4(-) action was confirmed: siRNA-mediated knockdown of vsp1S4(-) in infected cells increased VP7 expression (Fig 4E and 4F). Furthermore, wild-type vsp1S4(-) (pUC57-OpIE2-rORF1) and its transmembrane-intact variant (rORF1-EGFPmut) suppressed VP7, whereas constructs with a disrupted ORF (rORF1mut-EGFPmut) or empty vectors had no effect (Fig 4G and 4H). Furthermore, knockdown of endogenous rORF1 or expression of the rORF1 mutant significantly increased viral titers compared with the control group, with no significant difference between these two rescue groups; the uninfected group maintained low baseline titers. These results demonstrate that rORF1 suppresses BmCPV replication, and this inhibitory effect is abolished by rORF1 knockdown or mutation (S5C Fig).

Collectively, these results establish vsp1S4(-) as an inhibitor of BmCPV replication. Its coordinated expression with viral RNA, specific suppression of VP7 synthesis, and attenuation of viral spread reveal a mechanism whereby antisense-encoded microproteins modulate dsRNA virus infection, potentially acting as a feedback regulator to fine-tune viral progeny production.

### vsp1S4(-) interacts with NSP8 to antagonize RNAi-mediated antiviral responses to suppress their replication

Previous studies established that vsp1S4(-) localizes to the plasma membrane and serves as a structural component of the BmCPV virion. To define the functional role of the plasma membrane-localized virion-associated microprotein vsp1S4(-), we utilized AlphaFold 3 (AF3) to predict its interactions with BmCPV proteins. AF3 identified potential complexes between vsp1S4(-) and VP7, NSP8, or NSP9 (S6A-S6J Fig). Given that NSP8 functions as an RNA interference (RNAi) suppressor that promotes BmCPV replication [18], we prioritized the vsp1S4(-)-NSP8 interaction for mechanistic investigation.

**Fig 5:**
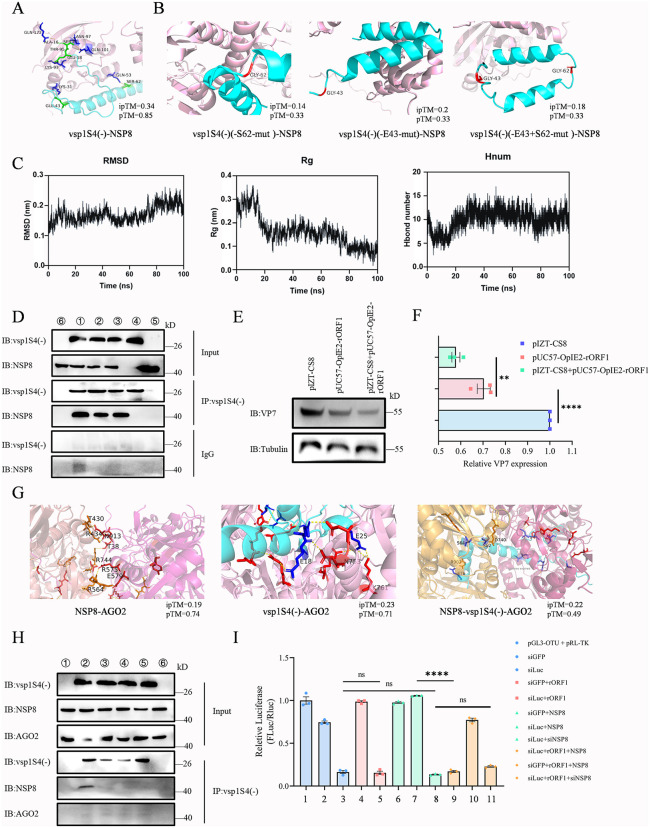
vsp1S4(-) antagonizes NSP8 to restore RNAi responses. (A-C) Structural and dynamic analysis. (A-B) (A) AF3-predicted binding: vsp1S4(-) S62/E43 ↔ NSP8 Q53/K31 (blue: vsp1S4(-); pink: NSP8). (B) Mutants vsp1S4(-) (S62G/E43G) were used to probe NSP8 binding. ipTM: Confidence analysis of residues. Interactions between different chains; pTM: The residues that may be analyzed experimentally can be predicted, and the global confidence analysis of the complex can also be predicted. (C) MD simulations of the vsp1S4(-)-NSP8 complex. The root mean square deviation (RMSD), rotational radius (Rg), number of hydrogen bonds. (D-F) Functional validation. (D) vsp1S4(-)-NSP8 interaction confirmed by Co-IP. BmN cells were co-transfected with NSP8 expression plasmid and the indicated EGFP-tagged vsp1S4(-) variants. Cell lysates were subjected to IP using anti-vsp1S4(-) antibody, followed by Western blotting with anti-vsp1S4(-) and anti-NSP8 antibodies. Input samples (10% of lysate) confirmed expression of both proteins where applicable. IgG was used as a negative control. ⑥ vector: pUC57-OpIE2-EGFPmut co-transfected with pIZT-CS8; ① wild-type vsp1S4(-), rORF1-EGFPmut co-transfected with pIZT-CS8; ②-④: S62G and E43G: single point mutants; DM: S62G/E43G double mutant co-transfected with pIZT-CS8; ⑤ mut: start codon mutant (non-expressing); Vector: empty vector control. Data represent three independent experiments with consistent results. (E-F) Co-transfected of pUC57-OplE2-rORF1 and pIZT-CS8 reduces the expression of VP7. (G-I) vsp1S4(-) disrupts NSP8-AGO2 binding. (G) vsp1S4(-) disrupts the NSP8-AGO2 interaction during triple coexpression (AF3-predicted pairwise binding). Blue: vsp1S4(-); pink: AGO2; orange: NSP8. (H) The interaction between vsp1S4(-), NSP8 and AGO2. ① vector: pUC57-OpIE2-EGFPmut co-transfected with pIZT-CS8 and AGO2; ② wild-type vsp1S4(-), rORF1-EGFPmut co-transfected with pIZT-CS8 and AGO2; ③-⑤ S62G and E43G: single point mutants; DM: S62G/E43G double mutant co-transfected with pIZT-CS8 and AGO2. ⑥ mut: start codon mutant (non-expressing). Vector: empty vector control. Data represent three independent experiments with consistent results. (I) Dual-luciferase assay showing restored RNAi activity. BmN cells were transfected with pGL3-OTU (firefly luciferase), pRL-TK (Renilla luciferase), and the indicated combinations of siGFP, siLuc, rORF1 (vsp1S4(-)-EGFP), NSP8, or siNSP8. Firefly luciferase activity was normalized to Renilla luciferase activity (mean ± SD, n = 3). ****P < 0.0001; ns, not significant (one-way ANOVA with Tukey’s test). vsp1S4(-) alone did not affect RNAi (Group 5 vs. Group 3, ns), but fully restored RNAi suppressed by NSP8 (Group 9 vs. Group 7, ****P < 0.0001). Knockdown of endogenous NSP8 enhanced RNAi (Group 8 vs. Group 3, ns), and vsp1S4(-) provided no additional effect (Group 11 vs. Group 8, ns), confirming that vsp1S4(-) functions exclusively by antagonizing NSP8. siGFP and siLuc were used as controls. (****P < 0.0001, n = 3).

To elucidate the molecular mechanism by which vsp1S4(-) suppresses BmCPV replication, we first investigated its potential interaction with viral proteins. AlphaFold 3 (AF3) structure prediction identified a stable complex between vsp1S4(-) and the viral RNAi suppressor NSP8, with key interfacial residues S62 and E43 of vsp1S4(-) engaging Q53 and K31 of NSP8 (Fig 5A). Site-directed mutagenesis (S62G/E43G) was predicted to abolish complex formation, supporting the specificity of these residues for interaction (Fig 5B). Molecular dynamics (MD) simulations (100 ns, 300 K) further confirmed the stability of the vsp1S4(-)-NSP8 complex, as evidenced by low root mean square deviation (RMSD), stable radius of gyration (Rg), and persistent hydrogen bonding throughout the simulation (Fig 5C; S7A Fig). The interaction interface was further validated by molecular docking analysis, highlighting S62 and E43 as critical binding sites (S7B Fig). To experimentally validate the vsp1S4(-)-NSP8 interaction, we performed co-immunoprecipitation (Co-IP) assays. BmN cells were co-transfected with NSP8 and various EGFP-tagged vsp1S4(-) constructs, including wild-type (WT), single point mutants (S62G, E43G), double mutant (DM, S62G/E43G), a non-expressing start codon mutant (MUT), and an empty vector control. Immunoprecipitation with anti-vsp1S4(-) antibody followed by Western blotting revealed that WT vsp1S4(-) efficiently co-precipitated NSP8, whereas single mutations partially reduced binding, and the DM completely abolished interaction (Fig 5D). To exclude the possibility that the EGFP tag mediates non-specific interactions, we performed two controls: EGFP alone did not co-precipitate NSP8 or AGO2, and endogenous Co-IP using a vsp1S4(-)-specific antibody in BmCPV-infected cells confirmed that native vsp1S4(-) interacts with both NSP8 and AGO2 (S7C, S7D Fig). Reverse Co-IP using anti-NSP8 antibody confirmed these results (S7E Fig). Importantly, when vsp1S4(-), NSP8 and AGO2 were co-expressed, WT vsp1S4(-) co-precipitated both NSP8 and AGO2, whereas the DM failed to interact with either protein, indicating that vsp1S4(-) binds both NSP8 and AGO2 through the same critical residues (S7F, S7G Fig).

We next assessed the functional consequences of vsp1S4(-) expression on viral replication. Co-expression of vsp1S4(-) with NSP8 significantly reduced the level of the major capsid protein VP7 compared to NSP8 alone, whereas the DM and MUT mutants failed to suppress VP7 expression (Fig 5E, 5F; S7H, S7I Fig). These results indicate that the interaction with NSP8 is required for vsp1S4(-)-mediated suppression of viral protein synthesis.

Given the suppression of RNAi by NSP8 through binding to Argonaute 2 (AGO2) [18], we examined the ternary interplay involving vsp1S4(-), NSP8, and AGO2. AF3 structural predictions revealed that vsp1S4(-) competitively disrupts the NSP8-AGO2 interface (Fig 5G). Importantly, WT vsp1S4(-) co-precipitated both NSP8 and AGO2, whereas the DM failed to interact with either protein (Fig 5H). Notably, the presence of NSP8 reduced the vsp1S4(-)-AGO2 interaction, suggesting a competitive binding mechanism. These results collectively demonstrate that vsp1S4(-) directly interacts with NSP8 and AGO2 through residues S62 and E43.

To investigate whether vsp1S4(-) influences AGO2 function independently of NSP8, we performed dual-luciferase reporter assays. BmN cells were transfected with pGL3-OTU (firefly luciferase reporter), pRL-TK (Renilla luciferase internal control), and various combinations of siLuc (to induce RNAi), NSP8, vsp1S4(-), siNSP8, or siGFP (negative control) (Fig 5I). As expected, siLuc alone efficiently knocked down firefly luciferase activity (RNAi positive control). NSP8 suppressed RNAi activity, as evidenced by restored firefly luciferase expression. Strikingly, co-expression of vsp1S4(-) fully restored RNAi activity in the presence of NSP8, indicating that vsp1S4(-) antagonizes the RNAi suppressor function of NSP8. vsp1S4(-) alone did not affect RNAi activity, excluding a direct activating effect on AGO2. Knockdown of endogenous NSP8 by siNSP8 enhanced RNAi activity, and addition of vsp1S4(-) provided no further increase, confirming that vsp1S4(-) functions exclusively by antagonizing NSP8. The DM mutant lost the ability to restore RNAi, consistent with its failure to bind NSP8. Together, these data demonstrate that vsp1S4(-) suppresses BmCPV replication by disrupting the NSP8-AGO2 complex, thereby restoring host RNAi-mediated antiviral defense (Fig 5I).

## Discussions

The discovery of vsp1S4(-), an antisense-encoded microprotein in the BmCPV dsRNA genome, expands our understanding of viral microprotein functionality and challenges conventional views of dsRNA virus gene expression. While dsRNA viruses have historically been assumed to encode functional proteins exclusively on sense strands, with antisense strands primarily serving as template strands [9,24], our integrated omics approach revealed a hidden repertoire of microproteins, including vsp1S4(-), encoded by previously unannotated antisense sORFs. To directly address the question of how the antisense transcript encoding vsp1S4(-) is generated, we performed strand-specific Northern blot analysis using a DIG-labeled probe targeting the vsp1S4(-) coding region. A specific transcript was detected in BmCPV-infected cells, confirming that the S4 antisense RNA is indeed produced during infection. This finding aligns with emerging evidence of antisense coding potential in RNA viruses [2,9,25] and highlights the underappreciated complexity of dsRNA virus-host interactions.

vsp1S4(-) translation is driven by an internal ribosome entry site (IRES) in the S4 antisense strand, a mechanism critical for cap-independent translation in viruses and stress-responsive cellular mRNAs [9,19,25,26]. The identification of a functional IRES-like element at positions 630–646 nt, validated through mutational analysis and luciferase reporter assays, underscores the adaptability of dsRNA viruses to exploit the host translational machinery under diverse conditions. This phenomenon is particularly significant for BmCPV, as IRES-mediated translation may facilitate protein synthesis during host stress or suppression of the cap-dependent pathway, ensuring that viral replication proceeds efficiently [19]. The 630–646 nt element functions as a cis-acting RNA element capable of driving cap-independent translation in our experimental systems, but classic bicistronic assays are needed to formally prove IRES activity. In addition, the RNA pull-down coupled with LC-MS/MS identified 40 candidate RNA-binding proteins that may interact with the IRES-like 630 element. Future studies are required to validate these interactions and define their functional roles in cap-independent translation.

Paradoxically, vsp1S4(-) acts as both a structural capsid component and a negative regulator of viral replication. Its localization to the virion surface, confirmed by immunogold labelling, plays a role in capsid architecture; however, its overexpression reduces viral structural protein (VP7) synthesis and genome abundance. This apparent contradiction is resolved by its interaction with NSP8, a known RNA interference (RNAi) suppressor [18]. By disrupting the NSP8-Argonaute 2 (AGO2) complex, vsp1S4(-) reinstates host RNAi responses, effectively dampening viral replication. Using site-directed mutagenesis, we identified residues S62 and E43 of vsp1S4(-) as critical for its interaction with NSP8. The S62G/E43G double mutant completely lost the ability to bind NSP8 and failed to suppress VP7 expression. Furthermore, when vsp1S4(-), NSP8 and AGO2 were co-expressed, the double mutant also failed to co-precipitate AGO2, indicating that these same residues mediate interaction with both proteins.

The canonical function of NSP8 as an RNAi suppressor relies on its ability to sequester AGO2, a key effector of the antiviral RNAi pathway [18]. vsp1S4(-) disrupts this interaction through direct binding, as demonstrated by coimmunoprecipitation and AlphaFold 3 modeling, thereby restoring AGO2-mediated silencing of viral transcripts. To exclude the possibility that the EGFP tag used in co-immunoprecipitation experiments mediates non-specific interactions, we performed two controls: EGFP alone did not co-precipitate NSP8 or AGO2, and endogenous Co-IP using a vsp1S4(-)-specific antibody in BmCPV-infected cells confirmed that native vsp1S4(-) interacts with both proteins. These controls ensure the specificity of our conclusions.

Importantly, dual-luciferase reporter assays demonstrated that vsp1S4(-) alone does not affect RNAi activity, but fully restores RNAi suppressed by NSP8. Knockdown of endogenous NSP8 by siNSP8 enhanced RNAi activity, and addition of vsp1S4(-) provided no further increase, confirming that vsp1S4(-) functions exclusively by antagonizing NSP8 rather than by directly modulating AGO2 activity. This mechanism represents a novel regulatory loop: while NSP8 promotes replication by inhibiting RNAi, vsp1S4(-) limits excessive viral production by antagonizing NSP8, potentially serving as a feedback regulator to optimize progeny yield and evade hyperactivation of host defenses. This dynamic interplay highlights the evolutionary arms race between viruses and their hosts, where microproteins emerge as critical mediators of immune evasion.

Viruses encoding a self-limiting factor are not unprecedented. In the same BmCPV system, VSP59 suppresses viral replication by inducing apoptosis [20]. vsp21, translated from vcircRNA_000048, interacts with ubiquitin carboxyl-terminal hydrolase. This interaction activates the NF-κB/autophagy pathway, which in turn suppresses BmCPV replication. [7]. And Akirin hijacks a viral peptide vSP27, activating the ROS-NF-κB pathway against viral infection [27]. With their circular single-stranded DNA genomes, geminiviruses encode the V3 protein from a sORF that localizes to the Golgi apparatus and functions as an RNA silencing repressor [2]. Similarly, MAVI1, produced during vesicular stomatitis virus (VSV) infection, is located in the endoplasmic reticulum (ER) and interacts with MAVS to inhibit viral replication [28]. The Zika virus (ZIKV) employs a uORF that affects human brain organoids, modulating the virus’s growth, virulence, and tropism [11]. vsp1S4(-) adds a new example: it suppresses BmCPV replication by antagonizing the RNAi suppressor NSP8, thereby restoring host RNAi.

In summary, vsp1S4(-) is an antisense-encoded microprotein that acts as a negative regulator of BmCPV. It is translated via an IRES-like element, binds NSP8 and AGO2, and re-establishes RNAi by disrupting the NSP8-AGO2 complex. This work reveals that dsRNA viruses, like other viruses, can use small antisense-encoded proteins to fine-tune their replication.

## Materials and methods

### Cell culture

BmN cells were stored in the Molecular Biology Laboratory of Soochow University and maintained in TC100 culture medium supplemented with 10% (v/v) fetal bovine serum (FBS; BI) at 26°C.

### Sequence analysis

The ViralORFfinder platform [2] was a gift from Professor Xueping Zhou’s laboratory at Zhejiang University for the prediction of all small open reading frames (sORFs) in the 10 segmented dsRNA genomes of BmCPV. NCBI ORFfinder (https://www.ncbi.nlm.nih.gov/orfﬁnder/) was used to predict the open reading frames (ORFs) in the dsRNA genome of BmCPV, and TBtools (https://github.com/CJ-Chen/TBtools-II/releases) was used to visualize the results. To investigate the conservation of an ORF-encoded protein of choice, BLASTp was used to identify proteins with high identity (e-value ≤ 0.05). The amino acids were analyzed via ClustalW (https://www.genome.jp/tools-bin/clustalw) and visualized via Jalview (https://www.jalview.org/) and Weblog (https://weblogo.berkeley.edu/logo.cgi).

### Plasmid construction

The pUC57 vector was modified by inserting the *Orgyia pseudotsugata* nucleopolyhedrovirus (OpNPV) IE2 promoter (OpIE2) upstream of the multiple cloning site (MCS), resulting in the control plasmid pUC57-OpIE2. The following constructs were subsequently derived from this backbone: EGFPwt and EGFPmut. The wild-type EGFP sequence (LOC100313953) or its mutant variant (start codon ATGGTG mutated to ATTGTT) was cloned and inserted into the MCS, yielding EGFPwt and EGFPmut, respectively. pUC57-OpIE2-rORF1: The predicted rORF1 sequence encoding vsp1S4(-) from BmCPV S4(-) (GU323606.1) was inserted into the MCS. pUC57-S4(-): The full S4(-) fragment was cloned and inserted into the MCS of pUC57-OpIE2. pUC57-S4(-)-EGFP: Constructed by replacing the vsp1S4(-) ORF (nt 899–1135) in pUC57-S4(-) with EGFP via in-frame substitution. rORF1-EGFPmut: To facilitate detection of the small vsp1S4(-) protein, rORF1 (lacking a stop codon) was fused in frame to the start codon-mutated EGFPmut (ATG → ATT) and cloned and inserted into pUC57-OpIE2. Control mutants: Mutated rORF1 (start codon ATG → ATT) was cloned and inserted into pUC57-OpIE2 or fused to EGFPmut, generating pUC57-OpIE2-rORF1mut and rORF1mut-EGFPmut. The pIZT-CS8 vector was stored in our lab. All plasmids were commercially synthesized by Sangon Biotech (Shanghai) Co., Ltd.

### Prediction of domains or signals in protein sequences

TMHMM (http://www.cbs.dtu. dk/services/TMHMM/) and Softberry (http://www.softberry.com/) were used to predict transmembrane domains (TMs). The internal ribosome entry site (IRES) was predicted by IRESite (http://www.iresite.org/). N6-methyladenosine (m^6^A) was predicted by SRAMP (http://www.cuilab.cn/sramp). The interaction between vsp1S4(-) and NSP8 was predicted via Alphafold3 (https://alphafold.com/) and visualized via PyMOL (https://pymol.org/).

### Antibody generation

A polyclonal anti-vsp1S4(-) antibody was raised in rabbits immunized with a KLH-conjugated synthetic peptide (C-MLTKLSSLSTR-NH_2_, corresponding to residues 2–12 of vsp1S4(-)). The antiserum was affinity-purified via the immunogenic peptide coupled to SulfoLink resin (HUABIO).

### Confocal microscopy

GFP fluorescence was observed via a Leica TCS SP8 point-scanning confocal microscope (Leica, France).

### *In vitro* transcription

The T7 sequence (TAATACGACTCACTATAG) was added in front of the IRES primer (Table 2) for PCR. The product gel was recovered and incubated at 37°C for 2–3 h with a Beyotime (Shanghai, China) in vitro transcription kit. The DNA template was digested by adding DNase I in a metal bath at 37°C for 15 min. Then, the RNA was purified with phenol and chloroform, and BmN cells were cotransfected with PEI (Beyotime, Shanghai, China) reagent and BmCPV.

### siRNA synthesis

sirORF1 (sense, GGUUGUCGCUUCUGAACAAUA; antisense, UUGUUCAGAAGCGACAACCUG) siRNA targeted the 11th ORF of segment 4. siLuc (sense, CUUACGCUGAGUACUUCGATT; antisense, UCGAAGUACUCAGCGUAAGTT) and siGFP (sense, GGCUACGUCCAGGAGCGCACC; antisense, UGCGCUCCUGGACGUAGCCUU) siRNAs targeting the firefly luciferase gene (*luc*) and the green fluorescent protein-encoding gene (*gfp*), respectively, were synthesized by Gima Corporation (Shanghai, China). siRNA transfection was performed via polyethyleneimine (PEI; Polysciences).

### Dual luciferase reporter gene assay

To assess IRES-like element activity, BmN cells (1 × 10^5^ cells/mL, 1 mL) were co-transfected with 1 μg of either pIZT-T7-IRES-Luc or pIZT-T7-Luc (control), together with 0.1 μg of pRL-TK (Renilla luciferase, RLuc) as an internal control. Cells were harvested 48 h post-transfection and lysed with passive lysis buffer. Luciferase activity was measured using the Dual-Luciferase Reporter Assay System (Promega, Madison, WI, USA). The luciferase activity of 100 μg of protein per sample was determined via a GloMax Multi Jr (Promega, Madison, WI, USA). Firefly luciferase (FLuc) activity was normalized to Renilla luciferase (RLuc) activity to account for variations in transfection efficiency and cell viability.

To assess the effects of vsp1S4(-) on siRNA-induced RNAi, 2 μg of the pIZT-CS8 or pUC57-OpIE2-rORF1 plasmid was cotransfected with 2 μg of pGL3OTU, 0.2 μg of pRL-TK, or 1 µg of siLuc/siGFP into BmN cells. The cells collected 48 hours after transfection were used for the dual-luciferase reporter gene assay.

### RNA extraction and real-time PCR

We used a Beyotime (Shanghai, China) total RNA extraction kit for RNA extraction from BmCPV-infected cells. A total of 4 μg of total RNA was reverse transcribed into cDNA via one-step gDNA removal and cDNA synthesis supermix (TransScript, Beijing, China; AT311–02). Real-time PCR was conducted using PerfectStart Green qPCR SuperMix (TransScript, Beijing, China, AQ601–01-V2). The primers used are listed in Table 2.

### RNA sequencing

RNA sequencing was conducted by OE Biotech Co., Ltd. (Shanghai, China). In brief, total RNA was extracted from both infected and uninfected BmN cells using a Beyotime total RNA Extraction Kit (Shanghai, China) according to the manufacturer’s protocol. The quality and quantity of the extracted RNA were assessed via a NanoDrop spectrophotometer and an Agilent Bioanalyzer. Only samples with an RNA integrity number (RIN) greater than 7 were used for RNA sequencing. The RNA libraries were prepared via a commercially available library preparation kit (Illumina TruSeq RNA Library Prep Kit). This process involved the fragmentation of RNA, reverse transcription into cDNA, ligation of adapters, and amplification of the library fragments. The prepared RNA libraries were sequenced on a high-throughput sequencing platform (Illumina NovaSeq 6000) to generate paired-end reads. The raw sequencing data were first processed to remove low-quality reads, adapter sequences, and other contaminants. The cleaned reads were then aligned to the BmCPV genome via a suitable alignment tool (STAR, HISAT2). The raw data were deposited in the NCBI Sequence Read Archive (SRA) with accession number PRJNA1308017.

### Ribo-seq

Cycloheximide (MedChemExpress, New Jersey, USA) was added to the cell culture medium to a final concentration of 0.1 mg/mL, and the mixture was incubated for 2 min. Then, the medium was discarded, and precooled phosphate-buffered saline (PBS) (Solarbio, Beijing, China) (injected with cycloheximide at a final concentration of 0.1 mg/mL) was added to the wells. The mixture was rinsed gently once to absorb the liquid, and the mixture was washed with precooled PBS again. The cells were collected and sent to Bluescape (Hebei, China) for sequencing and analysis. The raw data were deposited in the NCBI SRA with accession number PRJNA1308098.

### Molecular dynamics

The dynamic interaction between VSP1S4 (-) and NSP8 was investigated using molecular dynamics simulations with GROMACS 2022.4 and the Amber14SB force field. The 3D structures of the proteins were obtained through homology modeling or AlphaFold prediction. The simulations were performed in a solvated environment at 300 K and 1 atm pressure after proper equilibration. The trajectories were analyzed for key parameters, such as RMSD, RMSF, Rg, SASA, and hydrogen bond formation, to understand the conformational changes and interaction patterns between the two proteins. These results provide insights into the dynamics of their interaction, potentially revealing their functional roles in BmCPV infection.

### Northern blot

Total RNA (20 μg) from BmCPV-infected (48 h, MOI = 2) or mock-infected BmN cells was separated on a 1.2% denaturing formaldehyde-agarose gel and transferred to a nylon membrane. A digoxigenin-labeled DNA probe (237 bp) targeting the vsp1S4(-) coding region (S4 antisense, nt 899–1135) was generated by Sangon Biotech (Shanghai, China) Life Sciences. Hybridization was performed at 68°C overnight. After stringent washing, the signal was detected using anti-DIG-AP antibody and CDP-Star chemiluminescent substrate. Endogenous 18S and 5S rRNAs visualized on the ethidium bromide-stained gel served as internal size references.

### Plasmalemma staining

To validate the localization of vsp1S4(-), BmCPV-infected cells were stained with 250 nM plasmalemma-1 (PM-1) (MedChemExpress, New Jersey, USA), a fluorescent dye that specifically labels the plasma membrane. The original PM-1 solution was diluted in culture medium to a final concentration ranging from 1:1000–1:10000. The cells were incubated with the diluted PM-1 solution for 20–30 minutes at room temperature. The fluorescence signals were then visualized via a Leica TCS SP8 point scanning confocal microscope with a proportional scale of 1:25 nm to capture the PM-1 fluorescence and confirm the localization of vsp1S4(-) at the cell membrane.

### Sucrose density gradient centrifugation

Viral particles were purified from the supernatants of BmCPV-infected BmN cells (MOI = 2, 5 × 10⁶ cells) through sequential centrifugation: clarification at 3,000 × rpm (5 min), followed by ultracentrifugation at 20,000 × rpm (3 h). Purified virions were resuspended in 2 M KCl to precipitate structural proteins, after which the pellet was washed in ice-cold PBS and recovered by ultracentrifugation (30,000 × rpm, 3 h). The resuspended sample was layered onto a discontinuous sucrose gradient (20%, 25%, 30%, and 35% w/v) and ultracentrifuged for density-based separation. Gradient fractions were collected for Western blotting analysis.

### Virus immunogold assay

Immunogold detection of viral antigens was performed by Biomisp (Wuhan, China) according to standardized protocols.

### Immunohistochemistry of cells

BmN cells transfected with pUC57-OpIE2 and pUC57-OpIE2-rORF1 were fixed via submersion in 4% paraformaldehyde (room temperature) for 40 min to 24 h, incubated with 0.2% Triton X-100 (Beyotime, Shanghai, China) for 10–20 min, and washed with PBS three times for 5 min each. Normal goat serum blocking solution was added dropwise for 20 minutes at room temperature. Afterward, the mixture was discarded without washing. A vsp1S4(-) antibody (1:100) was added, and the mixture was incubated overnight at 4°C. After incubation was complete, the mixture was washed three times with PBS, each time for 3 minutes. A goat anti-rabbit secondary antibody (1:200) was added, and the mixture was incubated for 20 min. After that, the mixture was washed with PBS three times, each for 3 minutes. DAB (HUABIO, Shanghai, China) color development was performed for 3 min, and reasonable control of the reaction time was used to prevent excessive staining. After the completion of staining, the mixture was rinsed with deionized water for 10 minutes. Finally, the mixture was subjected to hematoxylin staining, and after staining, it was rinsed with distilled water for 10 minutes.

### Protein extraction and Western blotting

Total protein was extracted from BmN cells or *Bombyx mori* midgut tissue via RIPA lysis buffer (Beyotime, Shanghai, China) supplemented with PMSF (1:100) to inhibit protease activity. The lysate was boiled with 5 × loading buffer (Beyotime, Shanghai, China) for protein denaturation. For immunostaining and Western blotting, custom-made rabbit polyclonal antibodies against vsp1S4(-) (HUABIO, Shanghai, China) were used at dilutions of 1:200 and 1:2000, respectively. The additional primary antibodies used were anti-GFP (1:5000, Proteintech, Wuhan, China), anti-VP7 (1:10,000, homemade), anti-NSP8 (1:5,000), anti-VP1 (1:5,000), and anti-IgG (1:5,000, Proteintech, Wuhan, China) antibodies. Horseradish peroxidase-labeled IgG secondary antibodies (Cell Signaling Technology, Danforth, Massachusetts, USA) were applied as the secondary antibodies. The protein signal in the membrane was visualized via an enhanced chemiluminescence system (Amersham Pharmacia Biotech, London, England). The internal reference protein α-tubulin was used.

### Sodium Dodecyl Sulfate-Polyacrylamide Gel Electrophoresis (SDS-PAGE)

Total proteins were extracted from the midgut tissues of silkworms, enriched via ultrafiltration (UFC9030, Merck, Darmstadt, Germany) to obtain 30 kDa microproteins, mixed with 2 × SDS uploading buffer (0.1 mol/L Tris-Cl, 0.2 mol/L dithiothreitol, 4% SDS, 20% glycerol, 0.2% bromophenol blue, and 4% β-mercaptoethanol), and incubated for 10 min in a water bath at 100°C. The supernatant was centrifuged at 12,000 × g for 1 min and then subjected to SDS-PAGE using a 5% concentrated gel and a 12% separated gel (Solepol, Beijing, China). Proteins separated on the gel were visualized via silver staining.

### Co-immunoprecipitation (Co-IP) assays

pIZT-CS8 and rORF1-EGFPwt; rORF1-EGFPwt and pGADT7-AGO2; pIZT-CS8, rORF1-EGFPwt, and pGADT7-AGO2 were cotransfected into BmN cells, total proteins were extracted, and the cells were incubated with the prepared mouse anti-NSP8 antibody or rabbit anti-vsp1S4(-) and rabbit anti-AGO2 polyclonal antibodies at 4°C overnight. The protein-antibody complexes were incubated with protein A + G (CWBio, Taizhou, China; CW0349S) for 2 hours at 4°C. The complex was gently washed six times with 1 × PBS, centrifuged to retain the precipitate, and then incubated in boiling water with 5 × SDS Sample Buffer (Beyotime, Shanghai, China; P0015) for 10 min. After centrifugation, the supernatant was used for Western blotting. Infected and uninfected proteins extracted from the midgut of silkworms were incubated with rabbit anti-vsp1S4(-) antibody or IgG, and after incubation with protein A + G, the proteins were separated via SDS-PAGE. LC-MS/MS (Shanghai Lu Ming Biotechnology Co., Ltd.) was used to extract and identify the differential bands.

### RNA Pull-down

5′-Biotinylated probes were synthesized by Sangon Biotech (Shanghai, China) Life Sciences. The experimental probe used was AGGGACAACAGATATA (IRES-like 630 sequence), and the control probe used was ACCGCGACCGCGACCGCG.

Total protein extracts were incubated with probes overnight at 4°C with rotation. Streptavidin-agarose beads (50 μL; Thermo Fisher) were added to each mixture and rotated at 4°C for 2 h. The beads were washed three times with 1 mL of lysis buffer (50 mM Tris-HCl (pH 8.0), 150 mM NaCl, 15 mM NaN_3_, 0.5% NP-40, 1 mM DTT, 1 mM PMSF, and 1 × protease inhibitor), resuspended in 40 μL of H_2_O, and mixed with 40 μL of 2 × SDS loading buffer. Protein complexes were resolved by 4–20% gradient SDS-PAGE and silver-stained. The gels were stored in 3% acetic acid at 4°C until analysis. Bands corresponding to candidate trans-acting factors were excised for mass spectrometry identification.

### Statistical analysis

All data are presented as mean ± SD from three independent biological replicates (n = 3). Statistical significance was determined by two-tailed Student’s t-test for two-group comparisons or one-way ANOVA with Tukey’s post-hoc test for multiple comparisons. P values < 0.05 were considered significant. *P < 0.05; *P < 0.01; ***P < 0.001; ****P < 0.0001, ns, not significant.

## Supporting information

S1 Fig Integrated discovery pipeline for BmCPV-encoded microproteins. RNA-seq identified 1,105 BmCPV-responsive genes. Ribo-seq detected 567 translated ORFs, of which 89% corresponded to annotated CDSs and 3% were uORFs or overlapping ORFs. Integration with viral ORF predictions generated 703 candidates. LC-MS/MS analysis of the < 30 kDa protein fraction identified 17 peptides matching Ribo-seq data, confirming translation of previously unannotated sORFs.
(TIF)

S2 Fig Further characterization of potential microproteins. (A) MS analysis of peptides identified from less than 14.4 kDa nonhomologous protein bands in the midgut tissues of BmCPV-infected and BmCPV-uninfected silkworms. (a) ORF65-S2(-); (b) ORF27-S3(-); (c) ORF43-S3(-); (d) ORF29-S4(-). (B) Ribo-seq read counts of the 4 nonhomologous proteins from the antisense strands. (a) ORF65-S2(-); (b) ORF27-S3(-), ORF43-S3(-); (c) ORF29-S4(-). Genomic RNA(-) organization is shown in blue. Ribo-seq reads mapped to antisense ORFs are indicated by pink peaks.
(TIF)

S3 Fig Biochemical characterization of antisense sORFs. (A) Predicted antisense sORFs were analyzed for isoelectric point (pI), transmembrane domain, internal ribosome entry site (IRES), and N6-methyladenosine (m^6^A) modification potential. (B-C) Sample information and mass spectrometry report of vsp1S4(-) antibody. (D) Softberry was used to predict the subcellular localization of vsp1S4(-).
(TIF)

S4 Fig Determination of the viral components of vsp1S4(-). (A)vsp1S4(-) expression was confirmed and increased dose-dependently. Cells were transfected with the pUC57-OpIE2-rORF1 plasmid or the pUC57-OpIE2 control vector. (B) Salt‑based fractionation of BmCPV virion‑associated proteins. (C-D) Western blotting of structural proteins and nonstructural proteins. Data represent three independent experiments with consistent results.
(TIF)

S5 Fig *In vitro* transcription verification of the activity of IRES-like 630. (A) Schematic of in vitro transcription verification of IRES-like 487/630/837. (B) Deletion/mutation effects of IRES-like on VP7 expression determined via *in vitro* transcription. (C) Verification of the effect of vsp1S4(-) knockdown on BmCPV infectious virus titer.
(TIF)

S6 Fig The interaction between vsp1S4(-) and viral proteins. (A-J) Interaction between vsp1S4(-) and BmCPV protein (structural proteins: VP1, RdRp, VP3, VP4, VP6, and VP7 and structural proteins: NSP5, NSP8, NSP9, and Poly) complexes predicted via AF3. pLDDT: Complex residue confidence level for the predicted structure, ranging from 0-100; the higher the score is, the higher the confidence level. pTM: Analysis of global confidence in predicted complexes. ipTM: Confidence analysis of residue interactions between different chains.
(TIF)

S7 Fig vsp1S4(-) inhibits viral replication by disrupting the interaction between NSP8 and AGO2. (A) MD simulation supports stable vsp1S4(-)-NSP8 binding. Root mean square fluctuation (RMSF), solvent accessibility surface area (SASA), and free energy landscape (FEL) indicate the stability of the complexes. (B) The molecular docking interface between vsp1S4(-) and NSP8. S62 and E43 were the the key sites on vsp1S4(-). (C-D) The effect of GFP on the localization of vsp1S4(-). (C) Co-transfection rORF1-EGFPmut, pIZT-CS8 and BmAGO2; (D) Co-transfection pUC57-OpIE2-EGFPwt, pIZT-CS8 and BmAGO2. (E) vsp1S4(-)-NSP8 interaction confirmed by Co-IP. IP was using anti-NSP8; (F-G) vsp1S4(-)-NSP8-AGO2 interaction confirmed by Co-IP. (F) The IP was using anti-NSP8, while the (G) was using AGO2. (H-I) The effects of different VSP1S4(-) mutants on the expression of VP7.
(TIF)

S1 Table The potential ORF in the sense and antisense-strand of viral RNA. (XLSX)

S2 Table Mass spectrometry (LC-MS/MS) analysis of microprotein-derived peptides. (XLSX)
