# Supplementary figures and images for: IRES-like element-mediated translation of vsp1S4(-) suppresses BmCPV replication via RNAi antagonism
Source: PLoS Pathog. 2026 Jul 24;22(7):e1014402. doi: 10.1371/journal.ppat.1014402 (PMC13421769; doi:10.1371/journal.ppat.1014402)

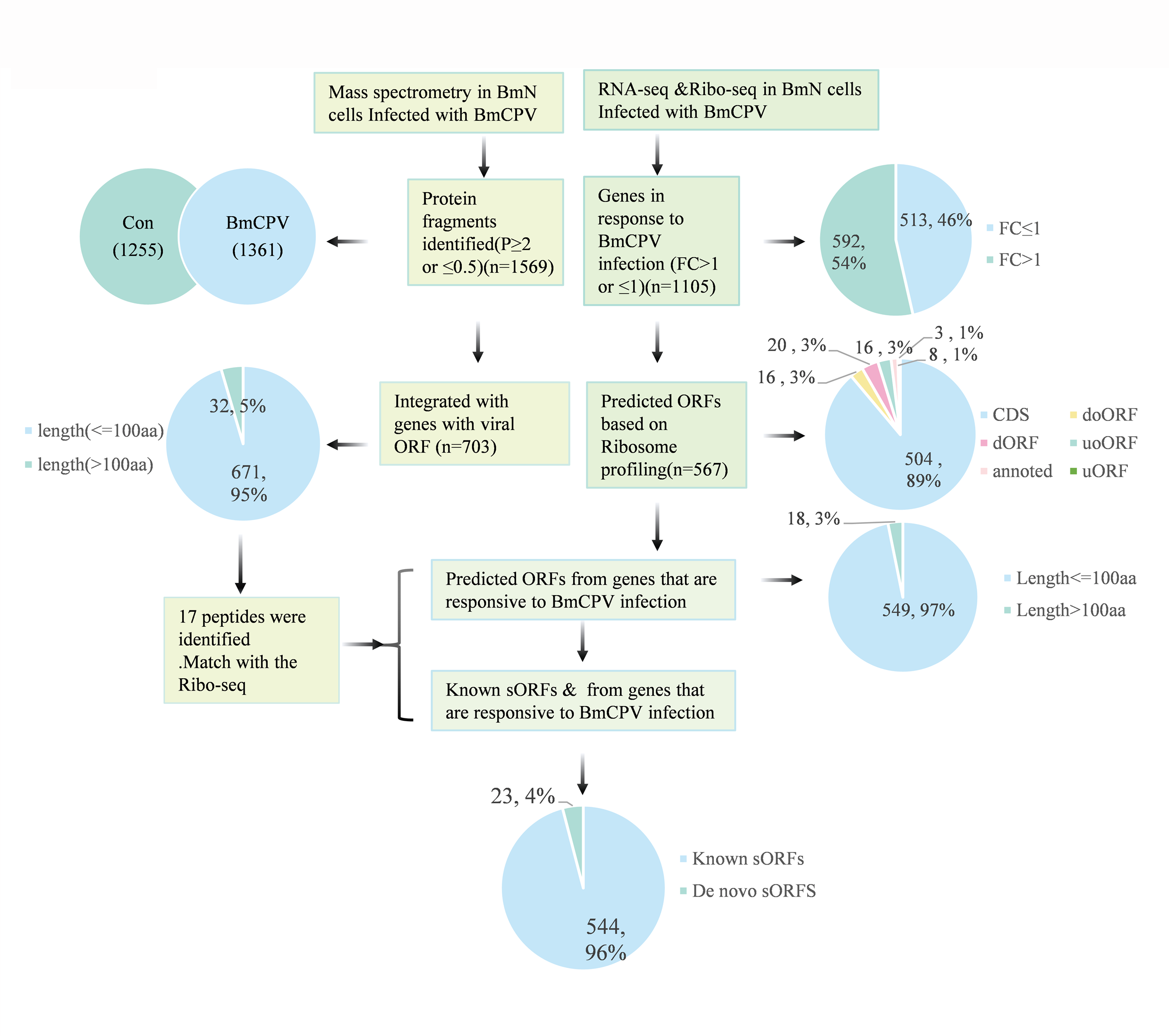

Supplement: S1 Fig — RNA-seq identified 1,105 BmCPV-responsive genes. Ribo-seq detected 567 translated ORFs, of which 89% corresponded to annotated CDSs and 3% were uORFs or overlapping ORFs. Integration with viral ORF predictions generated 703 candidates. LC-MS/MS analysis of the < 30 kDa protein fraction identified 17 peptides matching Ribo-seq data, confirming translation of previously unannotated sORFs. (TIF) [file ppat.1014402.s001.tif]

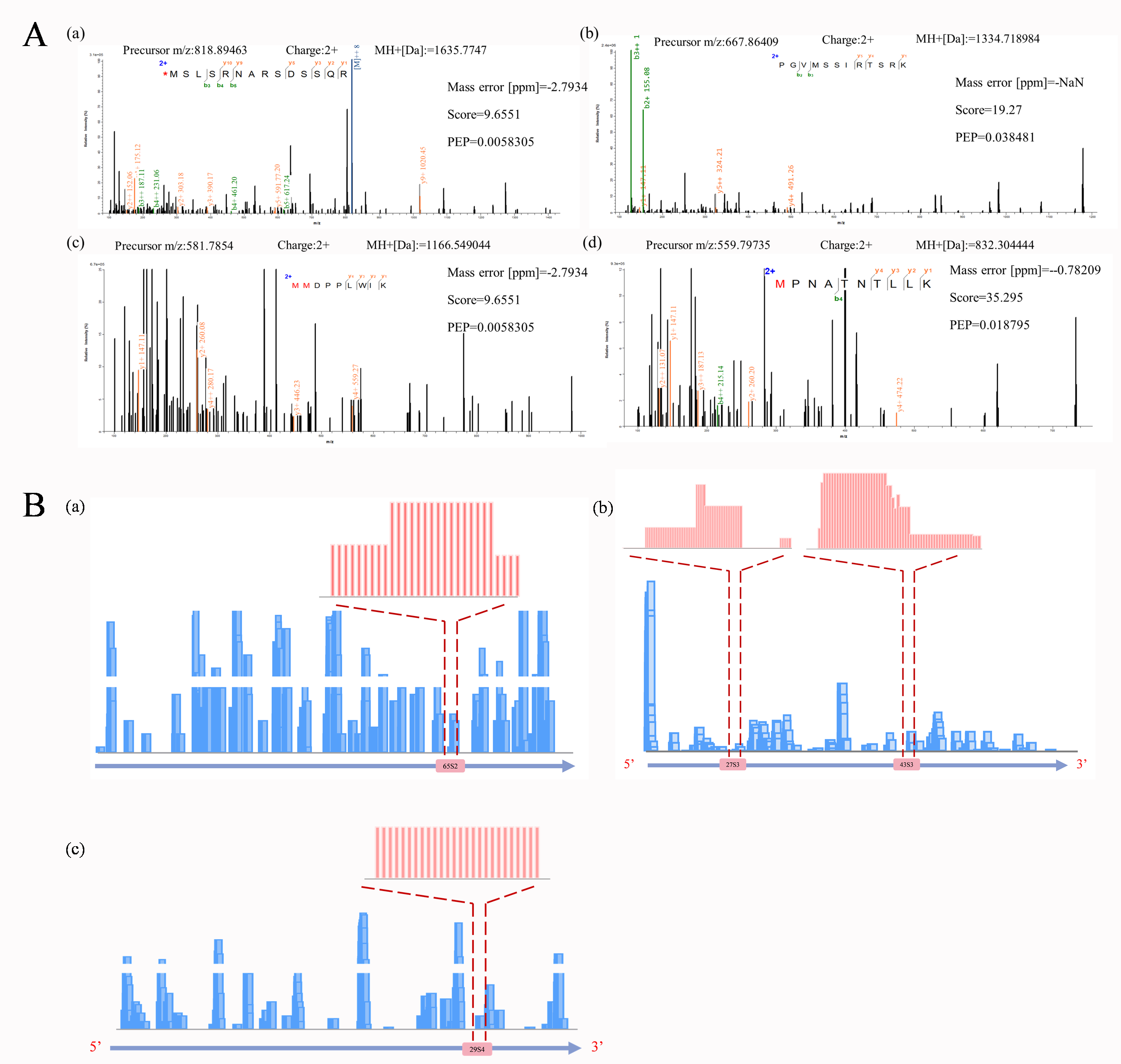

Supplement: S2 Fig — (A) MS analysis of peptides identified from less than 14.4 kDa nonhomologous protein bands in the midgut tissues of BmCPV-infected and BmCPV-uninfected silkworms. (a) ORF65-S2(-); (b) ORF27-S3(-); (c) ORF43-S3(-); (d) ORF29-S4(-). (B) Ribo-seq read counts of the 4 nonhomologous proteins from the antisense strands. (a) ORF65-S2(-); (b) ORF27-S3(-), ORF43-S3(-); (c) ORF29-S4(-). Genomic RNA(-) organization is shown in blue. Ribo-seq reads mapped to antisense ORFs are indicated by pink peaks. (TIF) [file ppat.1014402.s002.tif]

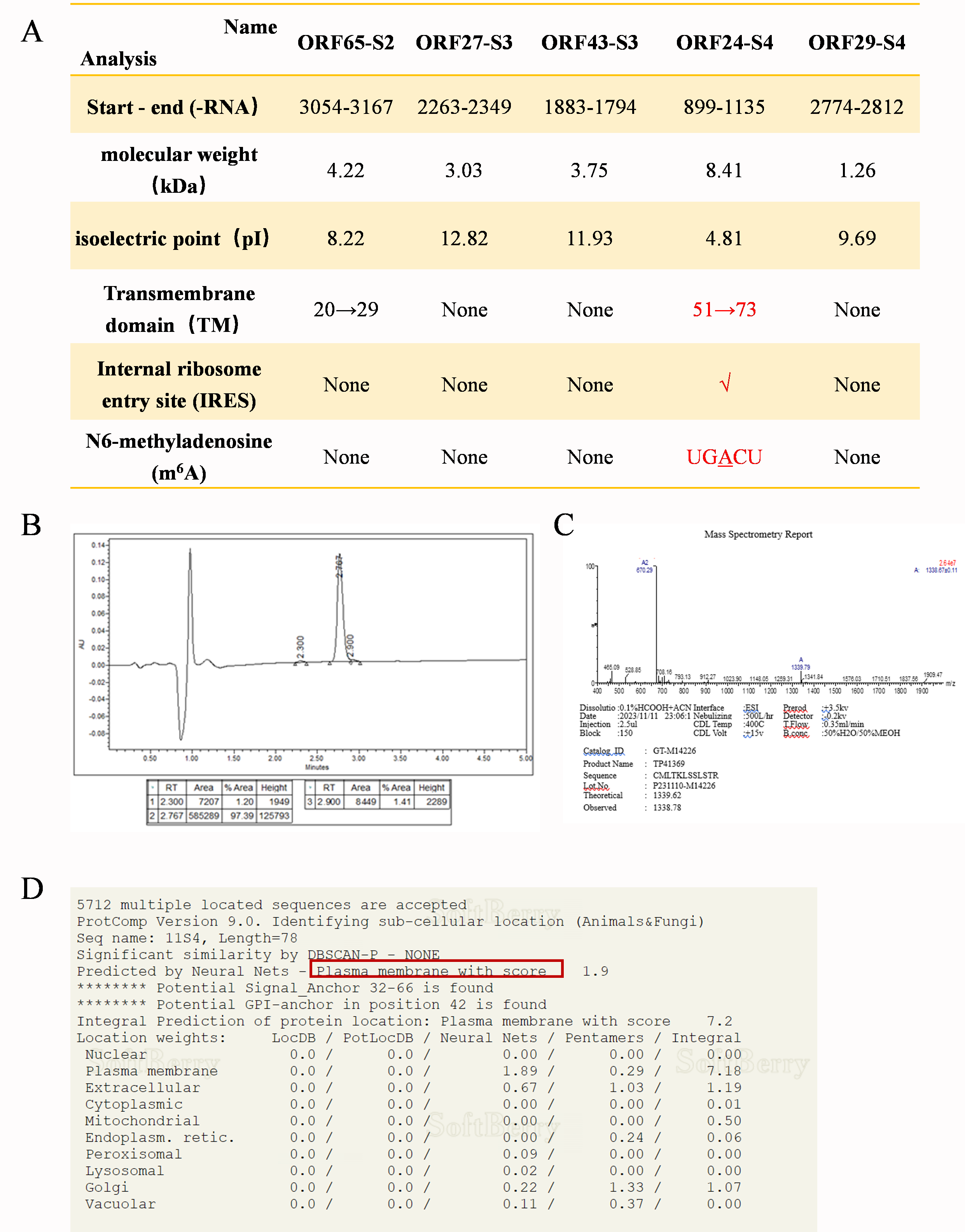

Supplement: S3 Fig — (A) Predicted antisense sORFs were analyzed for isoelectric point (pI), transmembrane domain, internal ribosome entry site (IRES), and N6-methyladenosine (m6A) modification potential. (B-C) Sample information and mass spectrometry report of vsp1S4(-) antibody. (D) Softberry was used to predict the subcellular localization of vsp1S4(-). (TIF) [file ppat.1014402.s003.tif]

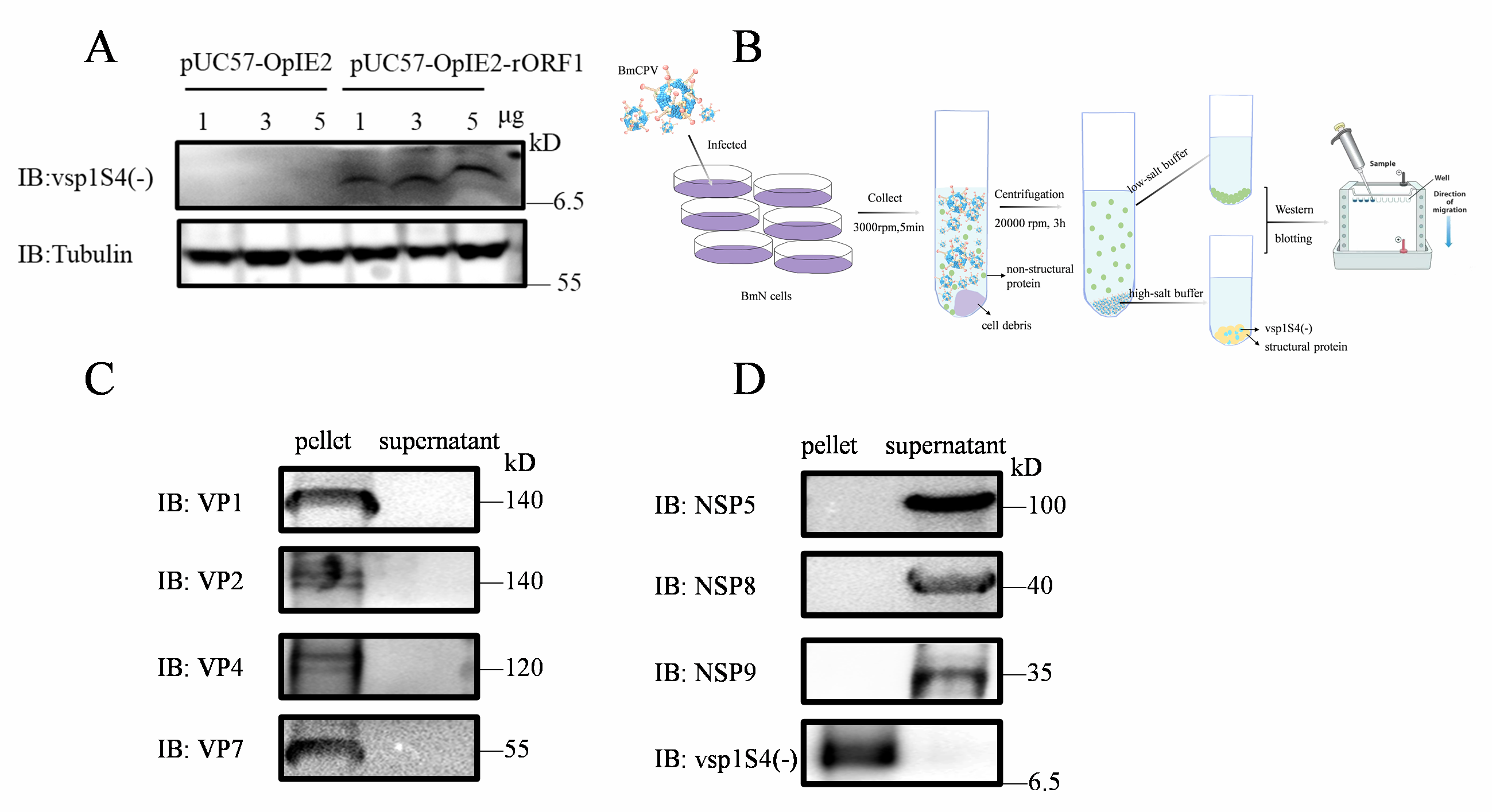

Supplement: S4 Fig — (A)vsp1S4(-) expression was confirmed and increased dose-dependently. Cells were transfected with the pUC57-OpIE2-rORF1 plasmid or the pUC57-OpIE2 control vector. (B) Salt‑based fractionation of BmCPV virion‑associated proteins. (C-D) Western blotting of structural proteins and nonstructural proteins. Data represent three independent experiments with consistent results. (TIF) [file ppat.1014402.s004.tif]

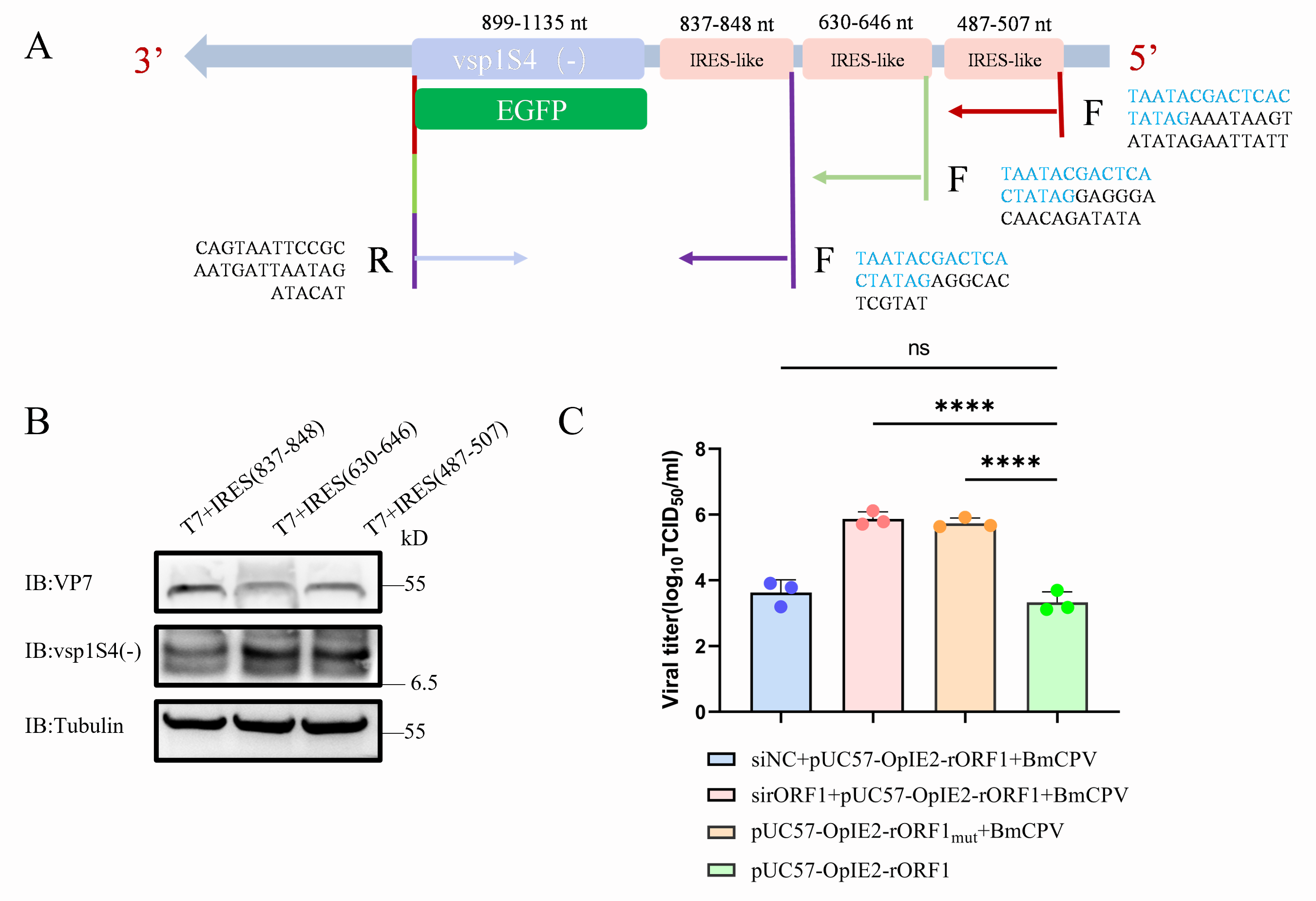

Supplement: S5 Fig — (A) Schematic of in vitro transcription verification of IRES-like 487/630/837. (B) Deletion/mutation effects of IRES-like on VP7 expression determined via in vitro transcription. (C) Verification of the effect of vsp1S4(-) knockdown on BmCPV infectious virus titer. (TIF) [file ppat.1014402.s005.tif]

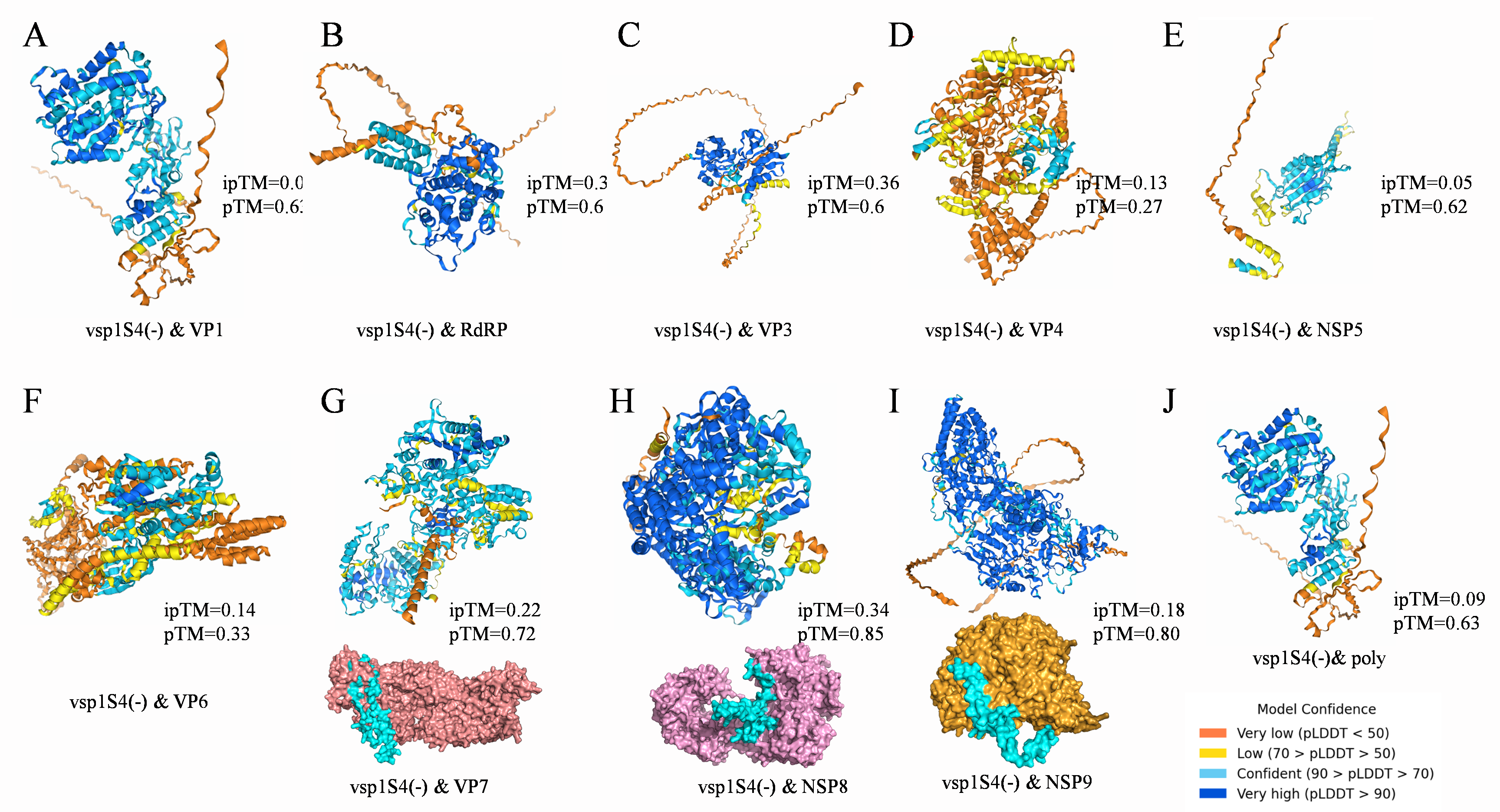

Supplement: S6 Fig — (A-J) Interaction between vsp1S4(-) and BmCPV protein (structural proteins: VP1, RdRp, VP3, VP4, VP6, and VP7 and structural proteins: NSP5, NSP8, NSP9, and Poly) complexes predicted via AF3. pLDDT: Complex residue confidence level for the predicted structure, ranging from 0-100; the higher the score is, the higher the confidence level. pTM: Analysis of global confidence in predicted complexes. ipTM: Confidence analysis of residue interactions between different chains. (TIF) [file ppat.1014402.s006.tif]

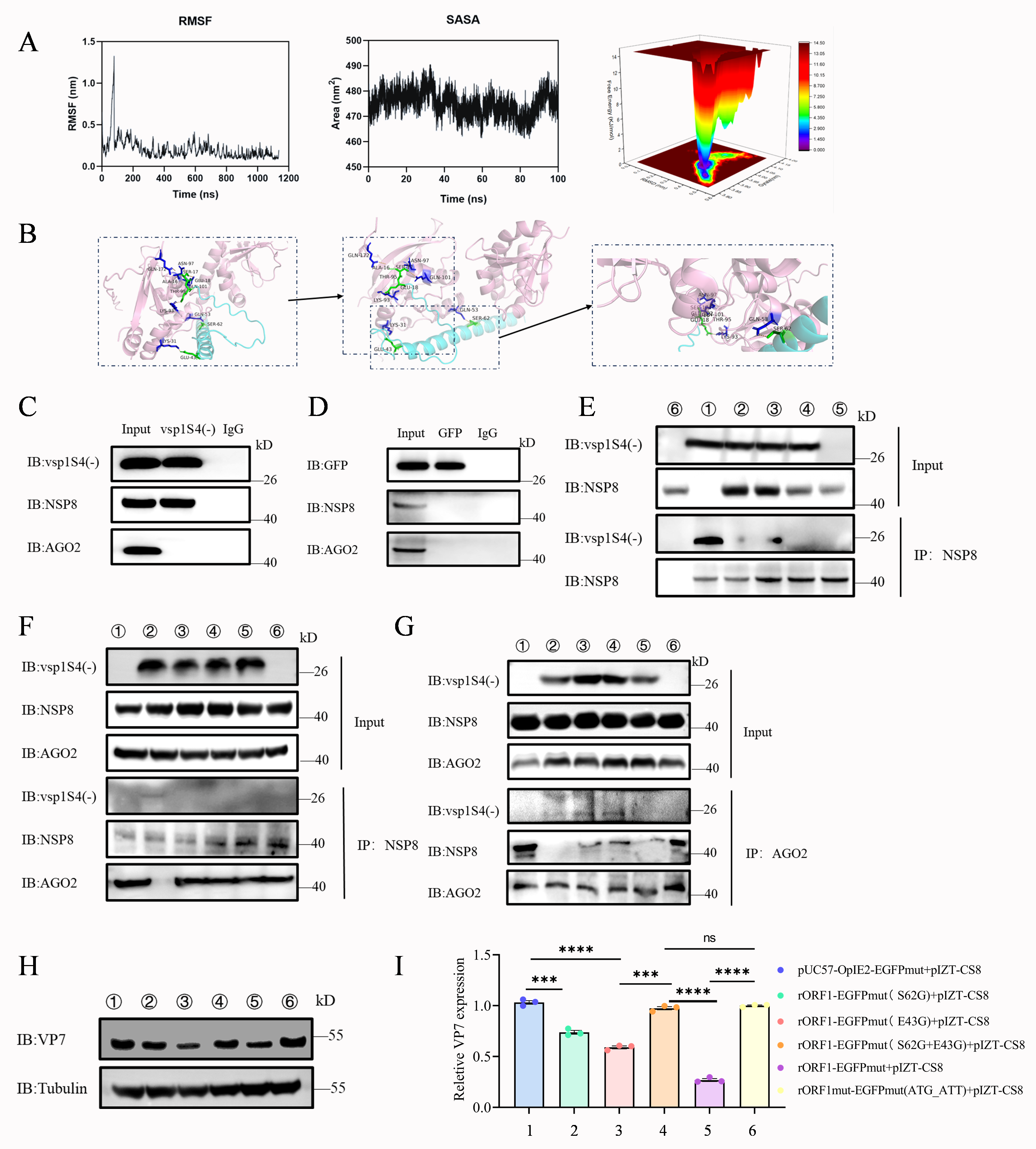

Supplement: S7 Fig — (A) MD simulation supports stable vsp1S4(-)-NSP8 binding. Root mean square fluctuation (RMSF), solvent accessibility surface area (SASA), and free energy landscape (FEL) indicate the stability of the complexes. (B) The molecular docking interface between vsp1S4(-) and NSP8. S62 and E43 were the the key sites on vsp1S4(-). (C-D) The effect of GFP on the localization of vsp1S4(-). (C) Co-transfection rORF1-EGFPmut, pIZT-CS8 and BmAGO2; (D) Co-transfection pUC57-OpIE2-EGFPwt, pIZT-CS8 and BmAGO2. (E) vsp1S4(-)-NSP8 interaction confirmed by Co-IP. IP was using anti-NSP8; (F-G) vsp1S4(-)-NSP8-AGO2 interaction confirmed by Co-IP. (F) The IP was using anti-NSP8, while the (G) was using AGO2. (H-I) The effects of different VSP1S4(-) mutants on the expression of VP7. (TIF) [file ppat.1014402.s007.tif]
